# Comment on “Recreational Athletes’ Use of Performance‑Enhancing Substances: Results from the First European Randomized Response Technique Survey”

**DOI:** 10.1186/s40798-023-00581-9

**Published:** 2023-05-29

**Authors:** Michael Petrou, Lambros Lazuras

**Affiliations:** 1 Cyprus Anti-Doping Authority (CyADA), Indoor Hall “Tassos Papadopoulos-Eleftheria”, Makarion Athletic Centre Avenue, Engomi, 2400 Nicosia, Cyprus; 2 Department of Psychology, Sociology, and Politics, Sheffield Hallam University, Sheffield, UK S10 5AY

Dear Editor,

We read with interest the manuscript “Recreational Athletes’ Use of Performance‑Enhancing Substances: Results from the First European Randomized Response Technique Survey” by Christiansen et al. [1]. We commend the authors’ effort to estimate the prevalence of doping use in recreational sport in Europe, but also observe important limitations, as follows.

## Methodological Limitations in the Assessment of Doping Prevalence

The term “doping” is clearly defined by the World Anti-Doping Code (the ‘Code’) [2] and international conventions [3, 4]. Likewise, the Prohibited List [5] published by the World Anti-Doping Agency specifies the substances and methods that are prohibited to use by athletes that fall under the scope of the Code and relevant anti-doping rules. Christiansen et al. [1] rightly acknowledged that their survey methods and results reflected recreational athletes *“own understanding of doping”*, and the relevant RRT question left the term “prohibited” open to subjective interpretation. This presents the following methodological limitations:

1. Recreational athletes do not necessarily fall under the scope of the Code and related anti-doping rules; thus, the term “prohibited” becomes elusive.
2. Certain prohibited substances (e.g., Ostarine) can be purchased as nutritional supplements in some countries. Without a clear definition of “prohibited” it remains unclear what the reported prevalence estimates reflect.
3. Recreational athletes may underreport doping use for motivational reasons, such as self-deceptive denial, as documented in previous substance use research [6, 7]. Participants may unintentionally engage in self-deception to preserve their sense of self-integrity [8]. John et al. [9] documented how these psychological processes lead to underreporting in RRT studies, and questioned the validity of resulting prevalence estimates.
4. Christiansen et al. [1] reported “*extraordinarily high*” (47%) instructional non-compliance – if almost half of the sample possibly did not understand the question, then the validity of the findings is seriously questioned.

Although a co-author of the Christiansen et al. duly acknowledged some of the abovementioned limitations in another publication using the same data [10], this was not the case in the said manuscript.

## Misguided Implications for Anti-Doping Policy

Based on their findings, Christiansen et al. [1] argued that doping in recreational sport is a “*myth*” and national anti-doping organisations (NADOs) should “*leave recreational athletes to themselves*”. Given the aforementioned limitations and associated validity concerns, these statements do not proportionally reflect the insights of the study and are misleading. They also neglect the findings of large international studies (e.g., Sagoe et al., meta-analysis of 187 studies) [11] that assessed the use of (unequivocally defined) prohibited substances, such as androgenic anabolic steroids and growth hormone, which are the most commonly used doping substances in recreational sport settings [12, 13]. Additionally, Christiansen et al. [1] explicitly advised NADOs to disregard doping in recreational sport. The correspondence between this argument and the reported findings is, at best, tenuous for all the reasons discussed above. Most importantly, this argument fundamentally contradicts calls by the international scientific community (e.g., Kanayama et al., [12]; McVeigh & Begley, [14]) and policy organisations, like the Council of Europe (European Sports Charter; and the Resolution adopted at the 2022 Conference of Ministers responsible for Sport) [15, 16] to address doping in recreational sport as a public health issue.

Overall, we are concerned that, unless the methodological limitations and tenuous inferences about anti-doping policy that are made by Christiansen et al. [1] are contextualised and re-evaluated, they can mislead NADOs and other relevant stakeholders, and potentially undermine the health of recreational athletes.

## Data Availability

Not applicable.

## Abbreviations

NADO: National anti-doping organisation
RRT: Randomized response technique
